# Molecular mechanism of autophagy in porcine reproductive and respiratory syndrome virus infection

**DOI:** 10.3389/fcimb.2024.1434775

**Published:** 2024-08-19

**Authors:** Xiaoyong Chen, Ziding Yu, Wenfeng Li

**Affiliations:** ^1^ Xingzhi College, Zhejiang Normal University, Jinhua, China; ^2^ College of Veterinary Medicine, China Agricultural University, Beijing, China; ^3^ College of Animal Sciences, Wenzhou Vocational College of Science and Technology, Wenzhou, China

**Keywords:** autophagy, porcine reproductive and respiratory syndrome virus, viral proteins, virus-host interaction, cellular factors

## Abstract

Porcine reproductive and respiratory syndrome virus (PRRSV), a significant pathogen affecting the swine industry globally, has been shown to manipulate host cell processes, including autophagy, to facilitate its replication and survival within the host. Autophagy, an intracellular degradation process crucial for maintaining cellular homeostasis, can be hijacked by viruses for their own benefit. During PRRSV infection, autophagy plays a complex role, both as a defense mechanism of the host and as a tool exploited by the virus. This review explores the current understanding of the molecular mechanisms underlying autophagy induction under PRRSV infection, its impact on virus replication, and the potential implications for viral pathogenesis and antiviral strategies. By synthesizing the latest research findings, this article aims to enhance our understanding of the intricate relationship between autophagy and PRRSV, paving the way for novel therapeutic approaches against this swine pathogen.

## Introduction

**Figure 1:**
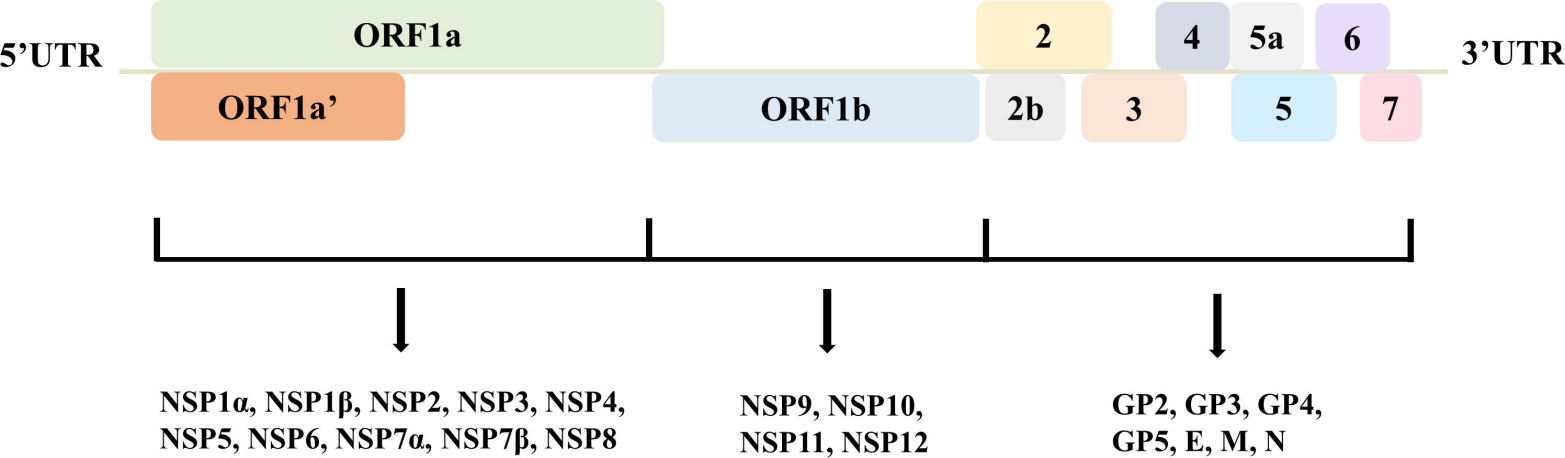
The genome structure of PRRSV. ORF1a and ORF1b encode nonstructural proteins 1-12, while ORF2-7 express structural proteins, including envelope (E), glycoproteins 2-5, membrane (M), and nucleocapsid (N). These proteins orchestrate to finish viral life cycle and infection.

Porcine reproductive and respiratory syndrome virus (PRRSV) is a single-stranded, positive-sense RNA virus belonging to the family *Arteriviridae* (Meulenberg, 2000). The genome of PRRSV is approximately 15,000 nucleotides long and encodes at least 11 open reading frames (ORFs) (Johnson et al., 2011). The PRRSV genome is organized into a 5’ untranslated region (UTR), followed by ORF1a, ORF1b, and ORFs 2 to 7 (Dokland, 2010). The 5’ UTR plays a crucial role in the viral replication cycle, while the 3’ UTR is involved in genome replication and transcription (Gao et al., 2012; Wei et al., 2018). ORF1a and ORF1b encode large polyproteins that are proteolytically processed into smaller nonstructural proteins essential for virus replication (Tang et al., 2016). ORFs 2 to 6 encode the viral structural proteins, including the envelope (E), the glycoproteins GP2, GP3, GP4, GP5, and the membrane protein (M). These proteins are involved in virus assembly and entry into host cells (Lunney et al., 2016). ORF7 encodes the nucleocapsid protein (N), which is the most abundant viral protein and plays a vital role in virus assembly by encapsulating the viral RNA genome (**Figure 1**). The N protein is highly immunogenic and is a major target for the host immune response (Chand et al., 2012). The glycoproteins, particularly GP5, play a crucial role in virus entry and are involved in interactions with the host cell receptors, facilitating virus internalization (Popescu et al., 2017). The N protein, besides its structural role, also has been implicated in regulating host cell processes like apoptosis and immune evasion mechanisms (Zheng et al., 2024). This virus has evolved mechanisms to manipulate host cell organelles and immune responses, making it a challenging pathogen to control. Ongoing research aims to elucidate the precise functions of these encoded proteins and develop effective strategies to combat PRRSV infections in swine populations worldwide.

PRRSV represents a serious threat to the swine industry, causing significant harm to animal health and welfare, and resulting in substantial economic losses. PRRSV’s impact on sows is particularly devastating, as it can cause abortions, stillbirths, and the delivery of piglets with birth defects, severely undermining the reproductive capacity of affected herds (Lunney et al., 2016). This loss of viable offspring translates directly into reduced herd size and production output. In young pigs, PRRSV induces respiratory distress, often progressing to pneumonia, which significantly impairs growth rates and feed conversion efficiency (Salguero et al., 2015). Breeding pigs infected with PRRSV may experience temporary semen reduction and decreased sperm motility. The morbidity rate of lactating piglets aged 3 to 5 days is approximately 36%, with the mortality rate ranging from 30% to 100%. For nursery pigs at the age of 8-10 weeks, the morbidity rate reaches 65%. The onset of the disease in fattening pigs occurs between 120 to 180 days, and the morbidity rate reaches 50% to 80%, accompanied by a mortality rate as high as 50% to 100% (Sun et al., 2023; Li et al., 2024a). At this stage, pigs exhibit a transitory reaction upon infection, marked by a decrease in daily weight gain and an increase in the feed gain ratio, along with symptoms such as transient anorexia and mild coughing. In cases of secondary infection, symptoms can worsen, leading to poor growth or death.

The resulting mortality, especially in weanling piglets, further compounds the economic losses. The costs associated with treating sick animals, increased veterinary expenses, and the need for enhanced biosecurity measures all contribute to the financial burden imposed by PRRSV (Neumann et al., 2005). In the US, economic losses due to PRRSV infection have increased from about $560 million per year prior to 2005 to about $664 million per year in 2013 (Holtkamp et al., 2013). Moreover, the average loss per sow after a PRRSV outbreak is about 126 euros, which imposes a heavy financial burden on pig producers (Nieuwenhuis et al., 2012). In 2006, China experienced a large outbreak and epidemic of highly pathogenic PRRS (HP-PRRS) characterized by high morbidity, fever, and mortality, with the pathogen HP-PRRSV, a variant of the PRRSV type 2 (Tong et al., 2007). This HP-PRRS epidemic dealt a heavy blow to China’s pig farming industry that was unprecedented at the time, and considering the scale and level of domestic breeding in China, the annual economic loss caused by PRRS to China’s pig farming industry should be much greater than that of the United States. Since 2013, PRRS caused by NADC30-like strains began to appear and become prevalent in China, which triggered new concerns and causes huger economic burdens (Zhou et al., 2015; Li et al., 2016a). Notably, in 2020, a new 1-4-4 strain was discovered in the United States, which is clinically costly and can lead to sow mortality (Trevisan et al., 2021; Rawal et al., 2023). The long-term persistence of PRRSV within a herd leads to chronic infections, necessitating prolonged and costly disease management strategies. The viral ability to spread rapidly and shed for extended periods poses a constant threat to uninfected pigs, necessitating strict segregation and quarantine measures (Martín-Valls et al., 2018). The economic toll of PRRSV is not limited to direct production losses. It also encompasses indirect costs such as market disruptions, trade restrictions, and consumer confidence erosion due to concerns over disease outbreaks. The swine industry’s reputation can suffer, potentially leading to decreased demand for pork products. The economic losses resulting from this virus are multifaceted, including decreased production, increased healthcare and veterinary costs, long-term disease management expenses, and potential market impacts (Nieuwenhuis et al., 2012). PRRSV’s widespread and enduring effects underscore the critical need for effective prevention, control, and eradication strategies to mitigate its devastating consequences on the swine industry and beyond.

The prevention of PRRSV is crucial to maintain the health and productivity of swine herds. Key preventive measures include maintaining strict biosecurity protocols to minimize the risk of virus introduction (Thanawongnuwech and Suradhat, 2010). This involves controlling access to the farm, ensuring that visitors, vehicles, and equipment are properly sanitized before entering the premises (Backhans et al., 2015; Sykes et al., 2022). Regular disinfection of animal housing, feeding areas, and water sources is also essential. Vaccination against PRRSV is another important preventive measure (Nan et al., 2017). Vaccines are available that can help reduce the severity of the disease and lower the risk of outbreaks. However, it’s important to consult with a veterinarian to determine the most suitable vaccination program for the specific herd, as the effectiveness of vaccines can vary depending on the virus strain and the herd’s immune status. Additionally, proper herd management practices play a vital role in preventing the spread of PRRSV (Pileri and Mateu, 2016). This includes regular monitoring of the herd’s health status, prompt isolation and treatment of sick animals, and maintaining strict hygiene standards (Risser et al., 2021). Avoiding overcrowding and ensuring adequate ventilation in animal housing can also help reduce the risk of virus transmission. Furthermore, education and training of farm workers on PRRSV prevention and control measures are crucial (Wang et al., 2023). They should be aware of the signs and symptoms of the disease, and understand the importance of following biosecurity protocols. The prevention of PRRSV requires a multifaceted approach that includes strict biosecurity measures, vaccination, herd management practices, and education of farm workers (Du et al., 2017). By implementing these strategies, swine producers can reduce the risk of PRRSV outbreaks and protect the health and productivity of their herds.

Autophagy, a conserved catabolic process involved in the degradation and recycling of cytoplasmic components, plays a complex role in the interaction between cells and viruses (Viret et al., 2018). PRRSV infection has been shown to trigger autophagy in host cells, which can either support virus replication or contribute to antiviral defense mechanisms (Sun et al., 2012). During PRRSV infection, autophagy can be induced as a cellular stress response, aiding the virus in its replication cycle by providing necessary nutrients and energy. This process can also help the virus evade the immune system by concealing viral antigens within autophagosomes, thus avoiding detection by the host immune cells (Sun et al., 2024). However, autophagy can also function as a defense mechanism against PRRSV. By sequestering and degrading viral proteins or viral particles, autophagy can limit virus replication and spread. Additionally, autophagy can activate immune signaling pathways, leading to the production of antiviral cytokines and interferons, which further inhibit virus replication. Understanding this complex interaction is crucial for developing effective strategies to combat PRRSV infection, as modulating autophagy pathways could potentially offer new therapeutic approach. Further research into this intricate relationship could lead to novel treatment options for this pathogen.

## PRRSV infection induces autophagy

**Figure 2:**
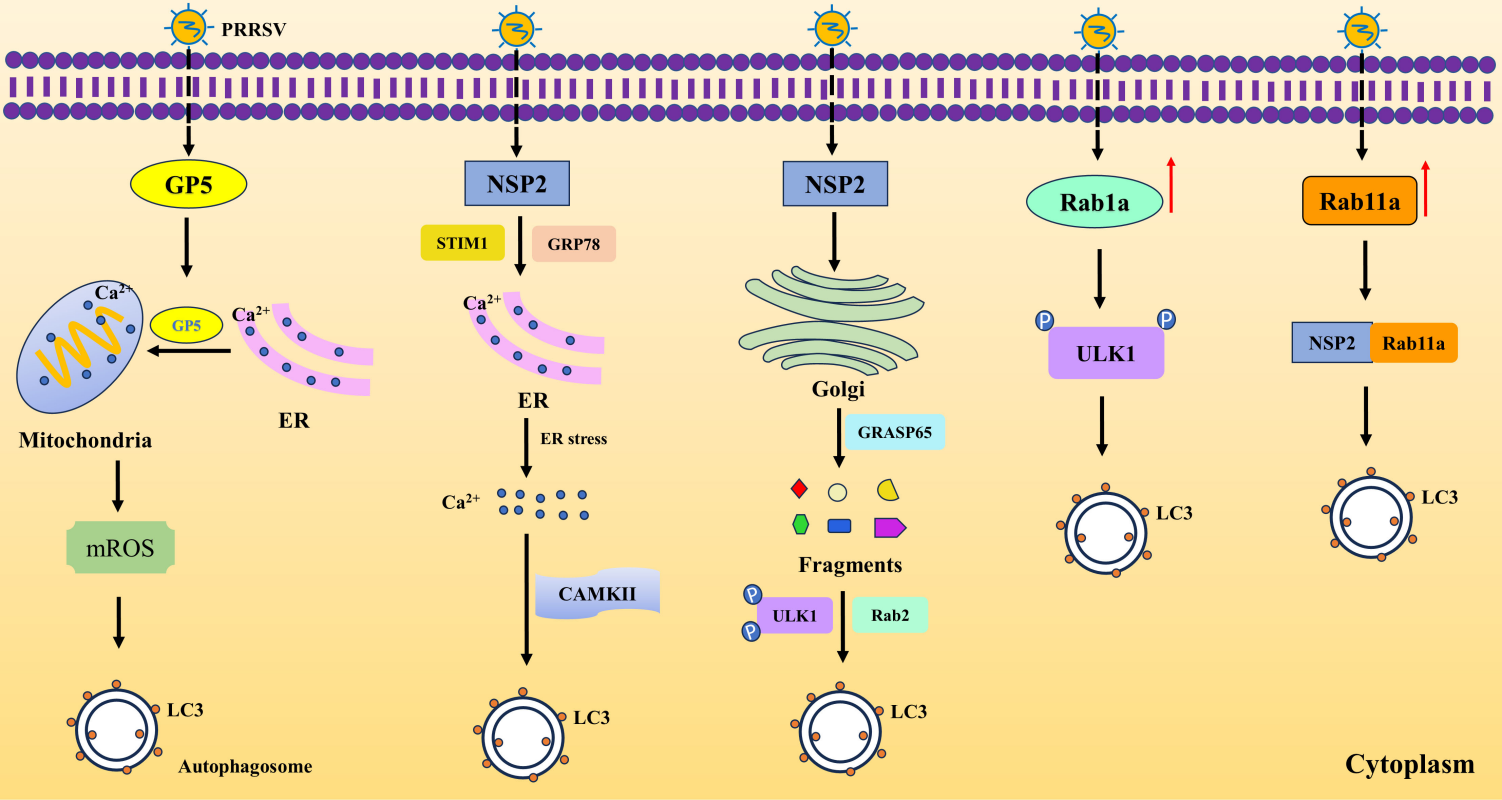
PRRSV infection induces autophagy via various pathways. PRRSV GP5 induces mitochondrial uptake of Ca^2+^ from ER, leading to mROS and autophagy. PRRSV NSP2 causes ER stress by interacting with STIM1 and GRP78, causing release of Ca^2+^ and autophagy via CAMKII. In the meanwhile, NSP2 degrades GRASP65, resulting in Golgi fragmentation and resulting in autophagy by phosphorating ULK1. Rab1a and Rab11a contribute to autophagy upon PRRSV infection. Rab1a promotes the phosphorylation of ULK1 and Rab11a interacts with NSP2 to cause autophagy.

The autophagy process induced by PRRSV begins with the virus entering the host cell and establishing infection. As the virus starts to replicate, it expresses viral proteins that interact with host cell machinery, initiating a cascade of signaling events. One of the key steps in autophagy induction by PRRSV involves the manipulation of calcium signaling within the cell. PRRSV infection can lead to an influx of calcium into the cytoplasm, either through the activation of calcium channels or the release of calcium from internal stores. This calcium influx triggers a signaling cascade that ultimately activates autophagy-related genes and proteins. However, there are two different mechanisms underlying the outcome by upregulation of calcium. Diao et al. found that PRRSV disrupts calcium homeostasis to activate calmodulin-dependent protein kinase-II (CaMKII) mediated autophagy, boosting viral replication. PRRSV induces endoplasmic reticulum (ER) stress, leading to calcium influx and release into the cytoplasm and viral NSP2 protein interacts with stromal interaction molecule 1 (STIM1) and glucose-regulated protein 78 (GRP78), key players in this process (Diao et al., 2023). While Zhang et al. discovered that PRRSV GP5 induces mitochondrial calcium uptake from ER, resulting in mitochondrial reactive oxygen species (mROS) release, which in turn induces autophagy and alleviates nucleotide-binding oligomerization domain-like receptor family pyrin domain containing 3 (NLRP3) inflammasome activation for viral replication (Zhang et al., 2023). Intriguingly, PRRSV hinders ER-phagy by suppressing the ER-phagy receptor family with sequence similarity 134, member B (FAM134B). Mechanistically, PRRSV-induced miR-142-5p targets FAM134B, boosting virus replication. Both depleting FAM134B and overexpressing its mutant protein disrupt ER-phagy, aiding in PRRSV replication (Guan et al., 2022). In addition, PRRSV infection upregulates Rab1a, a GTPase linked to autophagy. Rab1a interacts with UNC-51-like kinases 1 (ULK1), enhancing its phosphorylation and promoting autophagy, which benefits PRRSV replication (Jiang et al., 2023a). Notably, PRRSV also induces autophagy by fragmenting the Golgi apparatus (GA). The NSP2 interacts with and degrades a key GA protein, causing fragmentation. This fragmentation frees a Ras-like protein to bind with and activate an autophagy-related kinase, enhancing autophagy and viral replication (Zhao et al., 2024). Additionally, PRRSV could induce autophagy by upregulating Rab11a and NSP2 interacting with Rab11a contributes to the formation of autophagy (Wang et al., 2017). These studies indicate that PRRSV can induce autophagy via different mechanisms to benefit its own replication (**Figure 2**).

As autophagy is induced, the host cells begin to form autophagosomes, double-membraned vesicles that engulf cytoplasmic components destined for degradation. Rab11a, involved in intracellular membrane trafficking, is elevated by PRRSV infection. Further analysis showed that Rab11a is required for the autophagosome maturation and autophagy beneficial for PRRSV replication (Wang et al., 2017). Additionally, Zhang et al. showed that NSP3 and NSP5 proteins are responsible for the induction of autophagosome (Zhang et al., 2019). Nevertheless, PRRSV appears to hijack this process, possibly by interacting with autophagy-related proteins or by modulating signaling pathways that regulate autophagy. The virus may even directly induce the formation of autophagosomes around viral replication complexes, using them as a protected niche for virus replication. Once autophagosomes are formed, they fuse with lysosomes to form autolysosomes, where the engulfed material, including viral components, is degraded. However, PRRSV appears to have evolved mechanisms to prevent the complete degradation of viral material within autolysosomes, allowing it to exploit autophagy for its own replication. For instance, Sun et al. found that PRRSV suppressed the fusion between autophagosomes and lysosomes, resulting in the accumulation of autophagosomes that may serve as replication site to promote PRRSV replication (Sun et al., 2012). Furthermore, Xu et al. observed that glycoprotein non-metastatic melanoma protein B (GPNMB) suppress PRRSV replication by inhibiting autophagosome-lysosome fusion (Xu et al., 2023). Of note, NSP5 could interact with syntaxin 17 (STX17), disrupting its interaction with synaptosomal-associated protein 29 (SNAP29) and impeding autophagosome-lysosome formation (Zhou et al., 2023).

The exact mechanisms by which PRRSV interacts with autophagy machinery are still being elucidated, but it is clear that the virus takes advantage of this cellular process to enhance its replication (Chen et al., 2012; Liu et al., 2012). However, there are host factors that may target autophagy to regulate PRRSV replication. For example, miR-204 was found to target and inhibit LC3B-mediated autophagy, thus negatively regulating PRRSV replication (Yao et al., 2023). This intricate relationship between PRRSV and autophagy underscores the viral adaptability and ability to manipulate host cell processes for its own survival. In summary, PRRSV induces autophagy through a complex mechanism involving calcium signaling and the manipulation of autophagy-related proteins and pathways. This process allows the virus to hijack autophagy for its replication, and persistence within the host.

## Autophagy associates with apoptosis during PRRSV infection

Apoptosis is a genetically programmed form of cell death essential for normal development and homeostasis (Elmore, 2007). It involves a cascade of molecular events leading to the orderly dismantling of the cell from within. Apoptosis plays a crucial role in eliminating damaged, infected, or unnecessary cells, preventing the spread of potentially harmful cells (Dejas et al., 2023). Both autophagy and apoptosis are highly regulated processes that can be triggered by various intracellular and extracellular signals. Under certain conditions, autophagy can precede or accompany apoptosis, suggesting a complex interplay between the two (D’Arcy, 2019). While autophagy primarily focuses on cellular survival and homeostasis, sustained or excessive autophagy can sometimes lead to apoptosis. Conversely, apoptosis can be triggered when autophagy fails to restore cellular homeostasis or when the cell receives a death signal (Sorice, 2022).

PRRSV infection has been shown to induce both apoptosis and autophagy in the thymus of piglets (Wang et al., 2015). *In vitro* studies indicated that PRRSV replication was suppressed in autophagy-deficient cells and enhanced when apoptosis was inhibited. Additionally, in autophagy-deficient cells, the inhibition of apoptosis led to a resurgence of PRRSV replication (Li et al., 2016c). Inhibiting autophagy with 3-MA boosted PRRSV-induced apoptosis, suggesting a link between these two processes. Zhou et al. found increased Bad (a pro-apoptotic protein) and Beclin1 (an autophagy regulator) expression in infected cells. Bad and Beclin1 formed a complex in infected cells, and Bad co-localized with Beclin1. Silencing Beclin1 reduced PRRSV replication and autophagy, while silencing Bad enhanced autophagy and viral replication. PRRSV phosphorylated Bad to promote cell survival. These findings indicate autophagy promotes PRRSV replication by delaying apoptosis via the Bad-Beclin1 complex (Zhou et al., 2016). Similarly, PRRSV-induced unfolded protein response (UPR), especially the protein kinase RNA-like ER kinase (PERK) pathway, promotes autophagy. In addition, it also induces ER stress-mediated apoptosis via C/EBP homologous protein (CHOP), caspase 3, and the Poly (ADP-ribose) polymerase (PARP). And inhibiting autophagy with 3-MA increases ER stress and apoptosis in infected cells (Chen et al., 2020).

## PRRSV targets immune factors by autophagy for evasion

Host innate immunity is the first line of defense against pathogens, involving various cellular and biochemical responses that are rapidly triggered upon encountering a foreign invader (Chen et al., 2018). PRRSV could be recognized by several pattern recognition receptors (PRRs), such as Toll-like receptor 3 and 7 (Xu et al., 2021; Huang et al., 2022). During PRRSV infection, PRRSV has also evolved multiple strategies to counteract host immune responses, among which autophagy is exploited by PRRSV to degrade host factors for immune evasion.

It was reported that PRRSV infection targets melanoma differentiation-associated gene 5 (MDA5). PRRSV infection enhances the interaction between sequestosome 1 (SQSTM1/p62) and MDA5 through phosphorylation modification of p62 by kinase CK2α and K63 ubiquitination of porcine MDA5 catalyzed by tripartite motif-containing 21 (TRIM21), triggering classic p62-mediated autophagy for the degradation of MDA5 (Sun et al., 2024). Additionally, NSP11 promoted the degradation of interferon-stimulated gene 15 (ISG15), partially by autophagy-lysosomal pathway, thus antagonizing its antiviral effects (Jiang et al., 2023b). DEAD-box helicase 10 (DDX10), an enzyme involved in RNA metabolism, has been found to impede PRRSV replication by innate signaling. PRRSV infection promotes the degradation of DDX10 through autophagy, reducing its antiviral activity. The viral E protein was identified to interact with DDX10 and induces its autophagy-dependent degradation, which is mediated by the cargo receptor SQSTM1/p62 (Li et al., 2023a). Intriguingly, B lymphocytes and T lymphocytes in the lymphoid nodes were significantly reduced after PRRSV infection, probably due to PRRSV-induced autophagy in thymic epithelial cells (Zhu et al., 2022).

The relationship between the host immunity and PRRSV is characterized by the viral ability to suppress the host natural defenses, allowing it to replicate efficiently and cause more severe disease. This understanding is crucial for developing effective strategies to combat PRRSV in the swine industry.

## PRRSV infection causes lipophagy

Lipophagy is a specialized form of autophagy, a conserved cellular degradation process that targets lipid droplets within cells (Shin, 2020). Lipid droplets are intracellular organelles that store neutral lipids, primarily triglycerides and sterol esters, and they play a crucial role in lipid metabolism and energy homeostasis (Olzmann and Carvalho, 2019). The process of lipophagy begins with the recognition of lipid droplets as cargo for autophagy. This recognition is mediated by specific autophagy receptors that bind to both the lipid droplet surface and the autophagy machinery. These receptors, such as SQSTM1/p62, neighbor of BRCA1 gene 1 (NBR1), and optineurin (OPTN), act as adaptors, bridging the lipid droplets to the autophagy-related proteins that assemble the autophagosome (Zhang et al., 2022). Once the lipid droplets are marked for degradation, autophagy-related proteins are recruited to form the phagophore, a cup-shaped double-membrane structure that engulfs the lipid droplet (Filali-Mouncef et al., 2022).

It was reported that the reduced levels of N-Myc downstream-regulated gene 1 (NDRG1) triggers lipophagy, boosting PRRSV replication by altering lipid metabolism. PRRSV infection lowers NDRG1 levels, while NDRG1 deficiency aids in PRRSV replication. NDRG1 deficiency decreases intracellular lipid droplets and boosts autophagy and free fatty acid production, whereas an autophagy inhibitor reverses these effects and hinders PRRSV replication. Thus, NDRG1 suppresses autophagy and lipid droplet degradation, thereby negatively regulating PRRSV replication (Wang et al., 2019). Yu et al. found that epigallocatechin gallate (EGCG), a green tea component, reduces the replication of both mild and severe PRRSV strains *in vitro*. Mechanistically, EGCG primarily affects virus replication and assembly, not its attachment, entry, or release. Interestingly, it also countered PRRSV-induced lipid droplet formation and lipid synthesis and reduced PRRSV-stimulated autophagy, crucial for virus growth (Yu et al., 2022). Most recently, Li et al. discovered that PRRSV utilizes RAB18 to induce lipolysis via chaperone-mediated autophagy. They further demonstrated that RAB18 interacts with heat shock protein member 8 (HSPA8) and perilipin-2 (PLIN2) for lipid droplet degradation and subsequent lipolysis, thereby promoting viral replication and assembly (Li et al., 2024b).

## PRRSV infection induces aggrephagy

Aggrephagy plays a crucial role in maintaining cellular homeostasis by selectively degrading protein aggregates. During virus infection, the cellular aggrephagy machinery may be activated to clear virus-induced protein aggregates, thus limiting the potential damage to the cell. By clearing protein aggregates, aggrephagy may indirectly affect virus replication by maintaining a healthy cellular environment. However, viruses may also evolve mechanisms to evade or hijack the aggrephagy pathway for their own replication and survival. Cao et al. investigated the role of NSP2 in the formation of aggresomes. They revealed that NSP2 induces autophagy, relying on aggresome creation to stimulate aggrephagy. NSP2 transmembrane and tail sections facilitate this process, while the cell protein 14-3-3ϵ binds to NSP2’s tail, influencing autophagy (Cao et al., 2020). Sun et al. discovered that NSP3 promoted the aggrephagic degradation of MDA5 through the aggrephagy receptor CCT2, thus counteracting host innate signaling (Sun et al., 2024).

## PRRSV infection triggers mitophagy

Mitophagy, a highly conserved cellular process in eukaryotic cells, refers to the selective degradation of mitochondria by autophagy. This mechanism is crucial for adjusting mitochondrial numbers and maintaining stable cellular energy metabolism. It critically regulates the replication of many viruses, including influenza virus (Wang et al., 2021), SARS-CoV-2 (Li et al., 2022), Zika virus (Ponia et al., 2021), and PRRSV (Zhang et al., 2018). PRRSV infection prompts mitochondrial fission and mitophagy, thereby reducing apoptosis in Marc145 cells. Specifically, PRRSV infection triggers the expression of dynamin-related protein 1 (DRP1), enhances the phosphorylation of DRP1 at Ser616, and promotes its relocation to mitochondria. Additionally, PRRSV boosts the expression of PTEN-induced putative kinase 1 (PINK1) and Parkin, stimulating the recruitment of Parkin to mitochondria. Inhibiting the expression of either DRP1 or Parkin was found to hinder PRRSV replication. Significantly, silencing either DRP1 or Parkin led to a notable increase in apoptotic signaling (Li et al., 2016b). These findings imply that PRRSV infection stimulates mitochondrial fission and mitophagy, contributing to virus replication by suppressing apoptosis.

## Host cells target viral proteins for antiviral defense

Upon invasion, host cells rapidly induce autophagic pathway to capture and degrade viral components, including viral RNA and viral proteins, aiding in the antigen-capturing in immune response to prevent the virus from replicating. Li et al. found that proteasome subunit β type 1 (PSMB1) PSMB1 acts as an anti-PRRSV host protein by degrading NSP12 via the selective autophagy pathway. PRRSV downregulates PSMB1 via interaction with transcription factor early B cell factor 1 (EBF1). PSMB1 regulates NSP12 degradation through autophagy, increasing intracellular autophagy levels when co-transfected with NSP12. Both molecules colocalize in lysosomes. The autophagy cargo receptor NBR1 and E3 ubiquitin ligase STIP1 homology and U-box-containing protein 1 (STUB1) interact with PSMB1 and NSP12 in NSP12 degradation. This degradation mainly depends on NSP12 ubiquitination at lysine 130 (Li et al., 2023b). The same family member, PSMB4, also serves to negatively regulate PRRSV replication. Overexpressing PSMB4 hinders PRRSV replication, while reducing PSMB4 has the opposite effect. PSMB4 promotes NSP1α degradation via autophagic lysosomal pathway and activates the NF-κB pathway, boosting type I interferon production (Yi et al., 2023).

## Conclusion and perspectives

Autophagy, an intracellular degradation process crucial for maintaining cellular homeostasis, plays a significant role in PRRSV infection. Through an extensive review of the literature, it becomes evident that autophagy is intricately involved in various stages of PRRSV infection, from viral replication to the modulation of the host immune response. PRRSV has evolved mechanisms to hijack the autophagy pathway, utilizing it for its own replication and survival within the host cells. This review highlights the complex interplay between autophagy and PRRSV, emphasizing its dual role in both promoting viral replication and potentially contributing to antiviral defense mechanisms. Furthermore, the manipulation of autophagy by PRRSV underscores the viral adaptability and survival strategies within the host. The activation of autophagy during PRRSV infection appears to be a double-edged sword, as it can either benefit the virus by providing a favorable environment for replication or hinder it by enhancing antiviral responses. The exact mechanisms by which PRRSV interacts with the autophagy machinery and the consequences of these interactions on viral pathogenesis remain areas of active investigation.

Marker genes are specific genetic sequences used to identify and track particular cellular processes or states and play a crucial role in studying autophagy. They associated with autophagy are typically those that are upregulated or downregulated during this process, serving as indicators of autophagic activity. These genes encode proteins that are either directly involved in the autophagy machinery, or are regulated by autophagy and can thus serve as proxies for autophagic flux. For example, PSMB1 and PSMB4 are from the same family and have antiviral effects on PRRSV replication via autophagy (Yi et al., 2023; Li et al., 2023b). These two proteins could serve as potential marker genes used for therapeutic treatment against PRRSV infection. Additionally, PRRSV NSP2 is a major factor that induces autophagy via various mechanism to benefit viral replication (Diao et al., 2023; Zhao et al., 2024). In view of this, small molecule inhibitors could be developed targeting NSP2 specific domains and structures to suppress the autophagy induced by NSP2, therefore inhibiting PRRSV infection. By monitoring the expression levels of these marker genes, researchers can gain insights into the activation status, progression, and regulation of autophagy in different cellular contexts and disease states.

In conclusion, autophagy plays a pivotal role in PRRSV infection, influencing both viral replication and the host immune response. A deeper understanding of these interactions could pave the way for novel therapeutic strategies targeting autophagy to combat PRRSV infection. Future research in this field holds promise for the development of more effective treatments against this virus.
